# miR-101 Suppresses Vascular Endothelial Growth Factor C That Inhibits Migration and Invasion and Enhances Cisplatin Chemosensitivity of Bladder Cancer Cells

**DOI:** 10.1371/journal.pone.0117809

**Published:** 2015-02-06

**Authors:** Ye Lei, Bin Li, Shiyu Tong, Lin Qi, Xiheng Hu, Yunbo Cui, Zengbo Li, Wei He, Xiongbing Zu, Zhi Wang, Minfeng Chen

**Affiliations:** 1 Department of Urology, Xiangya Hospital, Central South University, No. 87 Xiangya Road, Changsha, Hunan, 410008, China; 2 Department of Biochemistry and Molecular Biology, College of Basic Medicine, Key Laboratory of Medical Biotechnology of Hebei Province, Hebei Medical University, No. 361 Zhongshan East Road, Shijiazhuang, Hebei, 050017, China; 3 Department of General Surgery, Xiangya Hospital, Central South University, No. 87 Xiangya Road, Changsha, Hunan, 410008, China; 4 School of Life Sciences, Central South University, No. 110 Xiangya Road, Changsha, Hunan, 410008, China; University of South Alabama, UNITED STATES

## Abstract

**Background:**

The microRNA miR-101 is downregulated in several cancers, including bladder cancer. However, miR-101’s role in the invasion, metastasis, and chemosensitivity of bladder cancer cells remains unclear. This study was conducted to determine miR-101’s role on the lymphangiogenic molecule vascular endothelial growth factor C (VEGF-C) and their effects upon bladder cancer cell migration, invasion, and chemosensitivity to cisplatin.

**Methods:**

Two bladder cancer cell lines (T24 and 5637) and the tool cell line 293T were employed here. Bladder cancer cells were transfected with either a miR-101 overexpression vector or a scrambled-sequence lentivirus, both of which exhibited a high transfection efficiency. Non-transfection was used as a mock negative control. Wound healing and Transwell assays were performed to measure cell migration and invasiveness. A luciferase reporter assay was performed to validate miR-101’s interaction with VEGF-C’s 3′ untranslated region followed by RT-PCR and Western blot confirmation. An MTS assay was used to evaluate the cisplatin sensitivity of the cell lines.

**Results:**

miR-101 overexpression significantly inhibited the migration and invasiveness while significantly enhancing cisplatin sensitivity. miR-101 negatively regulated VEGF-C protein expression, and VEGF-C overexpression rescued the effects of miR-101 overexpression, indicating that miR-101 negatively regulates VEGF-C protein expression post-transcriptionally. miR-101 and VEGF-C interference independently enhanced cisplatin cytotoxicity in bladder cancer cells.

**Conclusions:**

miR-101 suppresses VEGF-C expression, inhibits cell migration and invasion, and increases cisplatin sensitivity in bladder cancer cells. This study provides new insight into miR-101’s role in bladder cancer and shows miR-101’s promise as a potential molecular target for bladder cancer.

## Introduction

Bladder cancer is the most common urinary tract malignancy, producing approximately 150,000 annual deaths worldwide [1] and is clinically characterized by its progression, recurrence, metastasis, and drug resistance [2, 3]. Despite aggressive chemotherapy, 10–20% of non-muscle-invasive bladder cancers ultimately progress to muscle invasive bladder cancers [4]. Enrichment of blood and lymph vessels in the urothelial lamina propria, blood vessel invasion, invasion depth, and regional lymph node status have been identified as independent prognostic factors of tumor-free survival post-cystectomy, with the majority of cases of stage II and above distally recurring with each additional positive lymph node increasing the mortality risk by 20% [5,6]. Although cisplatin is the first-line chemotherapy for advanced bladder cancer, the cisplatin/gemcitabine (GC) regimen has a median time-to-progression of only six months and has no effect on overall survival after radical cystectomy in high-risk patients [7]. Despite radical cystectomy or preoperative chemotherapy, uncontrolled lymphovascular invasion of bladder cancer continues to yield a poor clinical prognosis [8–10]. Therefore, further research on bladder cancer’s progression, recurrence, metastasis, and chemotherapeutic efficacy is needed.

MicroRNAs (miRNA) are phylogenetically-conserved small non-coding RNAs that negatively regulate targeted mRNA 3′ untranslated regions (3′UTR) in several cancers and have been increasingly identified as tumor suppressors or carcinogenic agents [11–13]. Moreover, multiple miRNAs have been previously associated with chemotherapeutic sensitivity in several cancer cell lines, including bladder cancer [14]. In particular, miR-101 has been well-established as a tumor suppressor with inhibitory effects on cellular proliferation, migration, and invasion. Specifically, lower miR-101 levels have been previously associated with bladder cancer [15] as well as prostate [16], ovarian [17], colorectal [18], liver [19], gastric [20], lung [21], breast [22], thyroid [23], and melanoma [24] cancers. With respect to bladder cancer, miRNA profiling of bladder transitional cell carcinoma (TCC) samples has revealed that miR-101 is downregulated in TCC, and that miR-101 inhibits cell proliferation and colony formation in TCC cell lines through directly repressing the histone methyltransferase EZH2 [15]. However, miR-101’s role (if any) in the invasion, metastasis, and chemosensitivity of bladder cancer cells remains unclear.

VEGF-C, a member of vascular endothelial growth factor (VEGF) family, is regarded as an important lymphangiogenic molecule and is known to increase the permeability of lymphatic vessels [25–27]. In cancer, VEGF-C is positively correlated with lymphatic spread in bladder cancer, enhances lung adenocarcinoma cell migration to lymphatic vessels, and modulates cisplatin resistance in gastric cancer cells [28,29,38]. Although bladder cancer is known to primarily spread through the lymphatics (with metastasis found most commonly in the regional pelvic nodes) [30], no study has yet identified a relationship (if any) between miR-101 with VEGF-C in bladder cancer cells. In the current study, miR-101 was shown to have an effect on bladder cancer cell migration, invasion, and cisplatin sensitivity by directly regulating its functional target VEGF-C. This data suggests that miR-101 may be a potential molecular target for bladder cancer therapy.

## Materials and Methods

### Cell Culture

The bladder cancer cell lines T24 and 5637 as well as the human embryonic kidney tool cell line 293T used in this study were purchased from the Type Culture Collection of the Chinese Academy of Sciences (Shanghai, China) and were maintained in RPMI-1640 (Gibco, Grand Island, NY, USA) and Dulbecco’s Modified Eagle Medium (DMEM; Gibco, Grand Island, NY, USA), respectively, supplemented with 10% fetal bovine serum (FBS) and 1% penicillin/streptomycin. All cell lines were incubated at 37- in humidified 5% CO_2_.

### Plasmid Construction and Lentivirus Preparation

A fragment of miR-101 was generated by using the following primers: sense, 5′-CGGGTACCGGTAGTCCTTCACTTCATGGGGAG-3′ and antisense, 5′-CGGAATTCAAA- AAACCCAGCCACCTGTTTCAC-3′ and inserted into the GV209 vector with a green fluorescent protein (GFP) reporter gene within the AgeⅠand EcoRⅠrestriction sites. The aforementioned miR-101 plasmid, pHelper1.0, and pHelper2.0 were co-transfected into 293T cells, and the lentiviral particle-enriched supernatant was obtained 48 h later. A scrambled sequence (5′-TTCTCCGAACGTGTCACGT-3′), which had no homology with the human gene, was used as a scrambled negative control. The VEGF-C cDNA was amplified using the following primers: sense, 5′-TCCGCTCGAGATGCACTTGCTGG- GCTTCTTC-3′ and antisense, 5′-ATGGGGTACCGTGCTCATTTGTGGTCTTTTCC-3′ and cloned into the GV147 vector with a red fluorescent protein (RFP) reporter gene containing the restriction enzyme sites XhoⅠand Kpn I. Non-transfection was used as a mock negative control (S1 Data). All sequences in the experiments were confirmed by sequencing as follows: VEGF-C siRNA, sense, 5′-AGAUCUGGAGGAGCAGUUAUU-3′ and antisense, 5′-UAACUGCUCCUCCAGAUCUUU-3′; negative control siRNA, sense, 5′-UACUCACUACUCGAGAUGCUU-3′ and antisense, 5′-GCAUCUCGAGUAGUGAGUAUU-3′. Then, the synthesized pair of complementary VEGF-C shRNA were placed into a pGenesil-1 vector (Genesil, Wuhan). Fluorescence-activated cell sorting (FACS) (Epics Altra Flow Cytosorter, Beckman, USA) was used for select GFP+ or RFP+ cells according to the manufacturer’s recommended procedure. The transfection efficiency of miR-101 (GFP) and VEGF-C (RFP) was calculated as follows: miR-101 transfection efficiency = GFP+ cell count/total cell count x 100%, and VEGF-C transfection efficiency = RFP+/total cell count x 100%. On this basis, the transfection efficiencies for miR-101 (GFP) and VEGF-C (RFP) were found to be 99% and 40%, respectively.

### RNA Isolation and Quantitative PCR

Total RNA was isolated using Trizol reagent (Invitrogen, Carlsbad, CA, USA) according to the manufacturer’s instructions. Isolated RNA was measured using a Nanodrop 1000 spectrophotometer (Thermo Scientific, DE, USA) with an OD_260_/OD_280_ ratio greater than 1.8 used for cDNA synthesis. The reverse transcription of miR-101 and its specific amplification were performed using a miRNA qRT-PCR Detection Kit (GeneCopoeia, MD, USA). U6 was used as an endogenous control. cDNA synthesis of VEGF-C was performed using a First-Strand cDNA Synthesis Kit (GeneCopoeia, MD, USA), and the real-time quantitative PCR (RT-qPCR) reaction of VEGF-C was performed using qPCR Mix (GeneCopoeia, MD, USA). GAPDH was used for VEGF-C template normalization. All primers were purchased from GeneCopeia. miR-101 and VEGF-C amplification were determined on a ROTOR-GENE 3000 platform. The target PCR threshold cycle (Ct) values were normalized by subtracting the U6 or GADPH Ct value, which provided the ΔCt value. Relative expression was calculated using the following equation: relative gene expression level = 2 ^-(ΔCt experimental group-ΔCt control group^).

### Western Blot Analysis

RIPA lysis buffer containing 1 mM PMSF was added to the cells in six-well plates and then centrifuged for 10 min at 4-. The total protein content of the supernatant was determined by the Lowry method. The protein sample was diluted, heated for denaturation, and then subjected to sodium dodecyl sulfate polyacrylamide gel electrophoresis (SDS-PAGE). After the proteins were transferred to a polyvinylidene difluoride (PVDF) membrane (Millipore, MA, USA), 5% skimmed milk powder was applied for 2 h at 37-. Then, 1:1000 dilutions of rabbit polyclonal primary antibody anti-VEGF-C (Cell Signaling Technology, MA, USA) or anti-β-actin (which served as a loading control; Bioworld Technology, Inc, USA) was incubated with the membrane overnight. The next day, the membrane was rinsed in TBST three times and then incubated with a fluorescent secondary anti-rabbit antibody for 2 h. The membrane was scanned by an infrared imaging system (Odyssey), and the band intensity was detected by Odyssey 3.0 analysis software.

### VEGF-C 3’UTR Wild Type and Mutant Type Construction and Luciferase Reporter Assay

TargetScan analysis was used to discover a seven-nucleotide complementary sequence between miR-101 and the VEGF-C 3′UTR. A wild type (wt) VEGF-C 3′UTR sequence including Xba1 restriction sites (5′-TCTAGAACGAACCGCCAGAAGGCTTGTGAGCCAGGATTTTCATATAGTGAAG- AAGTGTGTCGTTGTGTCCCTTCATATTGGAAAAGACCACAAATGAGCTAAGATTGTACTGTTTTCCAGTTCATCGATTTTCTATTATGGAAAACTGTGTTGCCACAGTAGAACTGTCTGTGAACAGAGAGACCCTTGTGGGTCCATGCTAACAAAGACAAATCTAGA-3′) in addition to a mutant type (mut) VEGF-C 3′UTR sequence with a completely different miR-101-binding sequence, were synthesized and each independently cloned into separate GV272 luciferase reporter vectors (Genechem, Shanghai, China). The previously described miR-101-overexpressing or scrambled-sequence plasmid along with either a wt or mut VEGF-C 3′UTR plasmid were co-transfected into 293T cells using Lipofectamine 2000 reagent (Invitrogen, CA, USA), creating four experimental groups. Then, 293T cell luciferase assays were performed to determine the interaction between miR-101 and the wt and mut VEGF-C 3′-UTR. Renilla and firefly luciferase activities were measured at 48 h post-transfection using the Dual-Glo Luciferase Reporter Assay System (Promega, Madison, WI, USA) according to the manufacturer’s protocols. The ratio of renilla-to-firefly luciferase value was recorded (S1 Table). Experiments were performed in triplicate wells, and the data represents the mean of five independent experiments.

### Wound Healing Assay

The target cells, including T24 and 5637 cells, were transfected with recombinant lentivirus (1×10^8^ transducing units/ml) and polybrene (5 ug/ml). After 48 h, a trypsinized single cell suspension was prepared and seeded in six-well dishes (5×10^5^/well) until the cells reached 80% confluence. Then, 1-ml sterile pipette tip was used to scrape a cross in the center (similar to a wound), rinsed with PBS three times, and the serum-free medium was replaced immediately. Cells were allowed to migrate for 24 h, and the scratches were carefully observed and photographed. Light microscopic and fluorescent images were captured with an Olympus IX71 microscope. The gap lengths were also calculated from the photomicrographs. Each cell line was measured in triplicate.

### Transwell Assay

A Transwell chamber (Corning Corporation, MA, USA) with 8-μm polyester membrane filter pores, was used for both the invasion assay and migration assay. The difference between the invasion assay and the migration assay was that the invasion assay used coated Matrigel (BD Bioscience, MD, USA) in the upper chamber, which simulated the characteristics of the extracellular matrix, while the migration assay did not. A total of 2×10^5^ lentivirus-infected cells were seeded into the upper chamber with 200 μl serum-free medium. A total of 500 μl medium containing 20% FBS was added to the lower chamber. After incubation for 24 h, the cells adhering to the lower surface of the membrane on the upper chamber were fixed in 90% ethanol for 10 min, stained with 0.1% crystal violet, counted in three random shots by microscopy, and photographed. The average number of cells in the three random shots were reported. All experiments were performed in triplicate.

### MTS Assay

The cell suspension was seeded in 96-well culture plates at a density of 4×10^3^ cells/well (0.2 ml/well) for 24 h before use. The culture medium was replaced with fresh medium containing cisplatin with different concentrations for 72 h. Next, MTS (20 μl/well) was added to each well, and the absorbance at 490 nm of each well was recorded with a universal microplate reader (Bio-Rad Hercules, CA, USA) after 3 h. Dose-inhibition rate curves were plotted according to absorbance values. The IC50 value (the 50% maximal inhibitory concentration of cisplatin) was also calculated. The assay was repeated in triplicate.

### Statistical Analysis

Results were expressed as means ± SDs. All data were analyzed using SPSS software version 21.0. Experiments were statistically analyzed by Student’s *t*-test or one-way ANOVA. *P*<0.05 was deemed to be statistically significant.

## Results

### miR-101 Inhibits Bladder Cancer Cell Migration and Invasion

**Fig 1:**
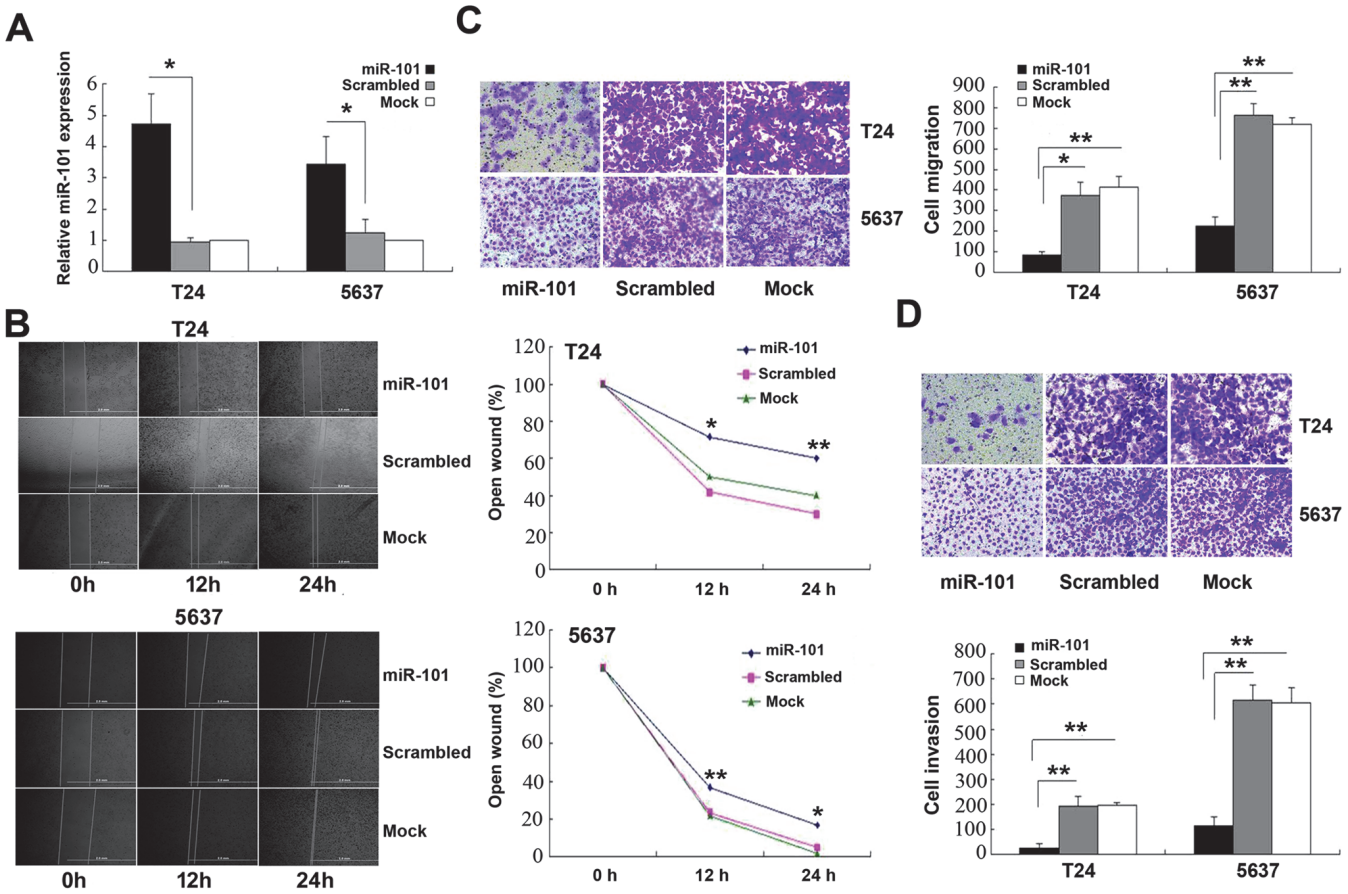
miR-101 Overexpression Suppresses Migration and Invasion of T24 and 5637 Bladder Cancer Cells. (A) Normalized miR-101 mRNA levels detected by RT-qPCR. (B) The open wound % (wound field_*final*_/wound field_*initial*_ x 100%) from the wound healing assay after transfection. (C) Representative images of migrated cells with the number of migrated cells per field. Note: T24 cells have a stronger affinity for crystal violent dye. Thus, although the image appears to show more abundant staining for T24 cells, the T24 cell counts were actually lower compared to those of 5637 cell. (D) Representative images of invasive cells penetrating the coated Matrigel and the number of invading cells per field. Data presented as means ± SDs. *p<0.05, **p<0.01.

Due to previous findings of lower miR-101 expression in bladder cancer [15,31], we constructed a miR-101 overexpression plasmid and packaged it into lentiviral vector. Bladder cancer cells were transfected with either a miR-101 overexpression vector or a scrambled-sequence lentivirus (scrambled negative control), both of which exhibited a high transfection efficiency (Fig. 1A). Non-transfection was used as a mock negative control. We then examined the cell migration and invasion ability of these bladder cancer cells. The wound healing assay and Transwell migration assay showed that cell migration was significantly inhibited by the miR-101-overexpressing lentivirus (Fig. 1B, 1C). The inhibitory effect of miR-101 on cell invasion was confirmed by the Transwell invasion assay. As a result, both T24 and 5637 miR-101-overexpressing cells in the bottom surface of the upper chamber exhibited dramatically inhibited invasiveness compared to their scrambled-sequence-transfected counterparts (Fig. 1D).

### miR-101 Directly Targets VEGF-C in 293T Cells

**Fig 2:**
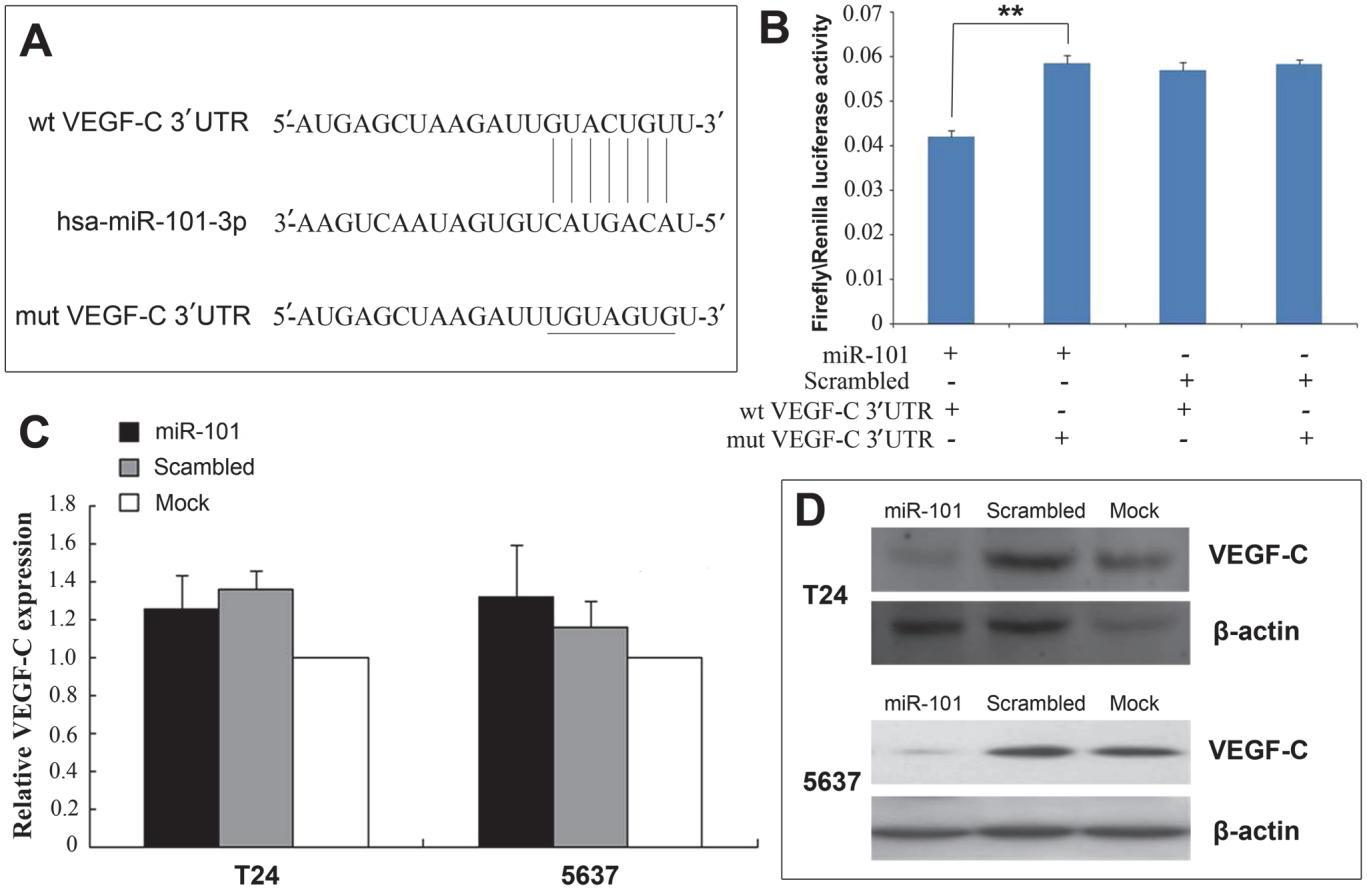
miR-101 Overexpression Directly Targets VEGF-C in T24 and 5637 Bladder Cancer Cells. (A) The potential binding sites for miR-101 on the seven bp-seed sequences of the 3′UTR region of VEGF-C. The mutant type was constructed at this seed region as underlined (wt: wild type, mut: mutant type). (B) Firely/Renilla luciferase activity after transfection with wt and mut VEGF-C 3′UTR and either miR-101-overexpressing vector or a scrambled-sequence vector. (C) VEGF-C mRNA expression measured by RT-qPCR in transfected cell lines showing no significant differences in VEGF-C mRNA expression across the three transfection states. (D) Western blot analysis of VEGF-C expression in transfected cell lines showing miR-101 overexpression significantly suppressing VEGF-C protein expression. Data presented as means ± SDs. **p<0.01.

To evaluate the molecular mechanism(s) of miR-101, TargetScan analysis found that VEGF-C is a potential target gene for miR-101, as there is a seven-nucleotide complementary sequence between miR-101 and the VEGF-C 3′UTR (Fig. 2A). Thus, a luciferase reporter assay showed that miR-101 upregulation significantly decreased the luciferase activity of wild-type VEGF-C but not the mutant VEGF-C (Fig. 2B). Then, RT-qPCR and Western blotting was used to assess the VEGF-C mRNA and protein levels, respectively (Fig. 2C, 2D). miR-101 overexpression significantly inhibited VEGF-C protein expression but did not significantly inhibit VEGF-C mRNA expression in both T24 and 5637 cells, suggesting post-transcriptional regulation of VEGF-C by miR-101.

### VEGF-C Overexpression Rescues miR-101’s Invasion Effects

**Fig 3:**
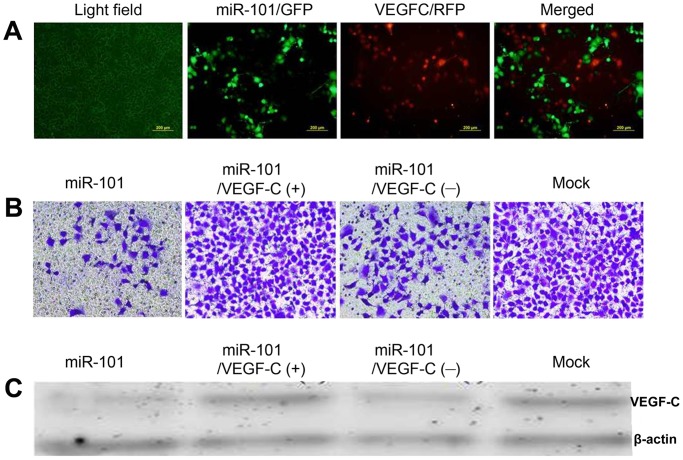
VEGF-C Overexpression Rescues miR-101 Overexpression Thereby Suppressing Invasion of T24 and 5637 Bladder Cancer Cells. (A) Representative images of bladder cancer cells simultaneously transfected with miR-101/green fluorescent protein (miR-101/GFP) and VEGF-C/red fluorescent protein (VEGF-C/RFP). (B) Representative images of invasive cells penetrating the coated Matrigel transfected with either miR-101, miR-101 and VEGF-C (miR-101/VEGF-C(+)), miR-101 and a negative control of VEGF-C (miR-101/VEGF-C(-)), or non-transfection (mock). (C) Western blot analysis validating VEGF-C protein expression in the transfected cell lines.

To verify the role of VEGF-C in the bladder cancer cell lines, we performed rescue experimentation (S1 Fig.). After T24 cells were transfected with the miR-101 lentivirus for 48 h, they were transiently transfected with a VEGF-C overexpression plasmid (Fig. 3A) to investigate the recovery of cell mobility. The Transwell invasion assay showed that the subsequent transfection of VEGF-C partially recovered the cells’ invasion ability compared to the VEGF-C negative control and non-transfected groups (Fig. 3B). As expected, the VEGF-C protein level was rescued (Fig. 3C). Thus, inhibition of bladder cancer cell invasion by miR-101 is likely a consequence of decreased VEGF-C expression.

### miR-101 Overexpression and VEGF-C Knockdown Independently Enhance Bladder Cell Sensitivity to Cisplatin

**Fig 4:**
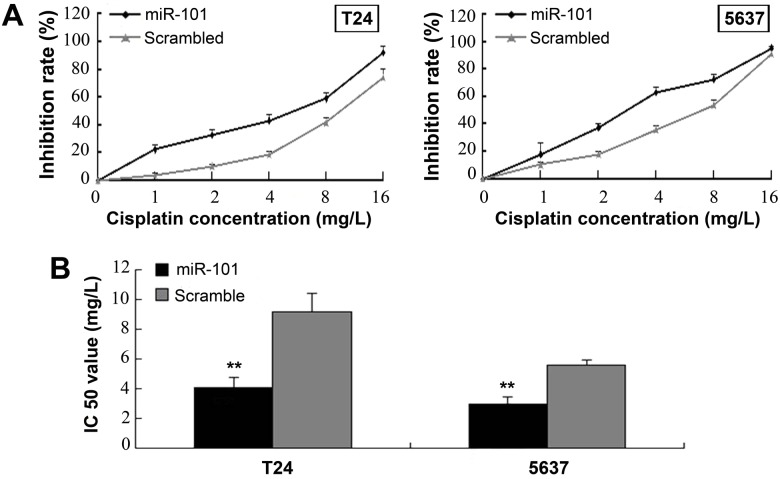
miR-101 Overexpression Enhances Cisplatin Sensitivity of T24 and 5637 Bladder Cancer Cells. (A) Plots of dose inhibition rate curves for cisplatin measuring cell counts after transfection with either miR-101-overexpressing or scrambled-sequence lentivirus. (B) IC50 values significantly reduced upon miR-101 overexpression. Data presented as means ± SDs. **p<0.01.

**Fig 5:**
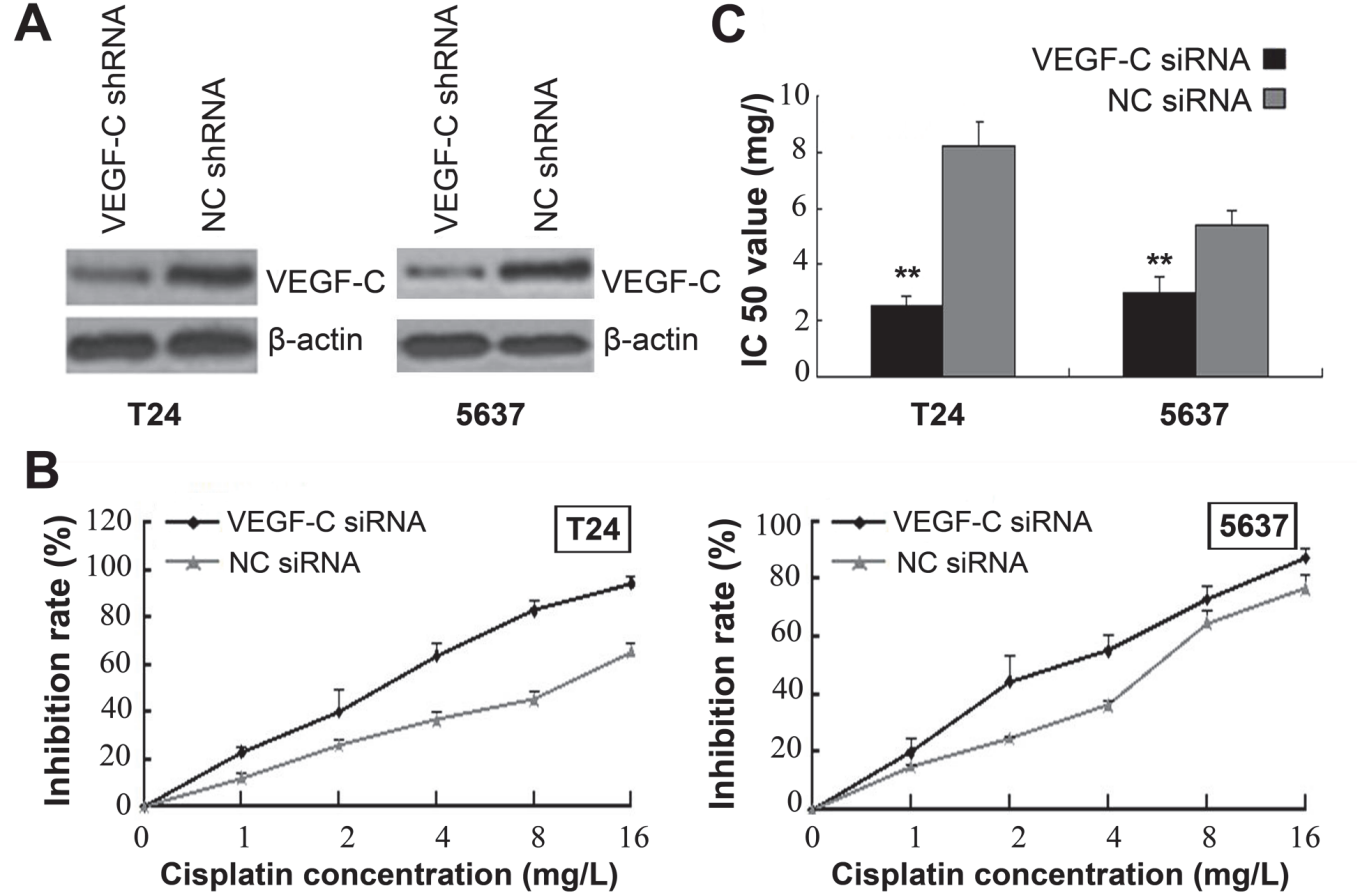
VEGF-C Knockdown Enhances Cisplatin Sensitivity of T24 and 5637 Bladder Cancer Cells. (A) Western blot analysis of VEGF-C expression in cell lines transfected with VEGF-C shRNA or control shRNA. (B) Plots of dose inhibition rate curves for cisplatin measuring cell counts after transfection with VEGF-C shRNA or control shRNA. (C) IC50 value significantly reduced after VEGF-C knockdown. Data presented as means ± SDs. **p<0.01.

miR-101 overexpression increased the sensitivity of both T24 and 5637 cells to cisplatin, with the IC50 value increasing from 4.06 ± 0.69 mg/l to 9.17 ± 1.24 mg/l and from 2.96 ± 0.46 mg/l to 5.56 ± 0.36 mg/l, respectively (Fig. 4A, 4B). In order to determine VEGF-C’s role in cisplatin sensitivity, a VEGF-C interference plasmid was constructed and transfected into the bladder cancer cell lines (Fig. 5A). The sensitivity of T24 and 5637 cells to cisplatin increased after VEGF-C interference, with the IC50 value increasing from 2.56 ± 0.31 mg/l to 8.23 ± 0.83 mg/l and from 3.01 ± 0.5 5mg/l to 5.42 x 0.52 mg/l, respectively (Fig. 5B, 5C). These results suggest that VEGF-C downregulation, as the downstream target of miR-101, enhances bladder cancer cell sensitivity to cisplatin.

### VEGF-C Overexpression Rescues miR-101-Induced Cisplatin Sensitivity

**Fig 6:**
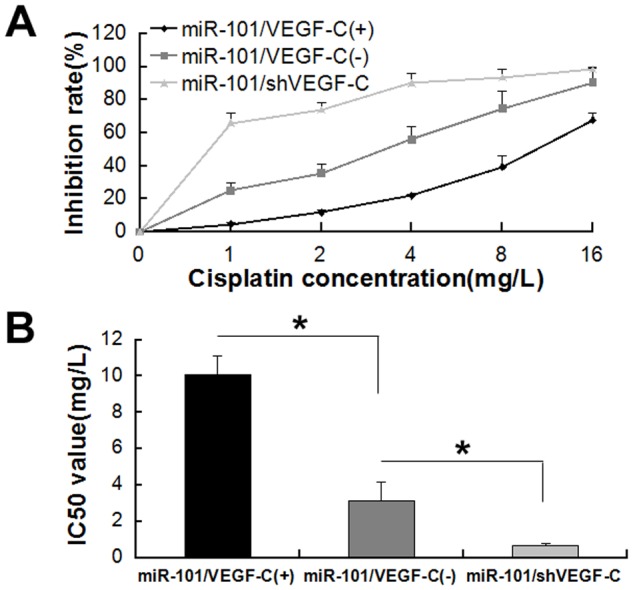
VEGF-C Rescues miR-101-Induced Sensitivity to Cisplatin. (A) Plots of dose inhibition rate curves for cisplatin measuring cell counts after transfection with either miR-101 and VEGF-C (miR-101/VEGF-C(+)), miR-101 and a negative control of VEGF-C (miR-101/VEGF-C(-)), or miR-101 and VEGF-C shRNA (miR-101/shVEGF-C). (B) The IC50 value was significantly increased after transfection with miR-101/VEGF-C(+).

After T24 cells were transfected with the miR-101 lentivirus for 48 h, they were then transiently transfected with either a VEGF-C overexpression plasmid, a VEGF-C control plasmid, or a VEGF-C shRNA plasmid. We found that VEGF-C overexpression partially recovered bladder cancer cell sensitivity to cisplatin compared the VEGF-C control with IC50 values of10.05 ± 1.05 mg/l and 3.14 ± 1.02 mg/l to 0.63 ± 0.16 mg/l, respectively (Fig. 6A, 6B). Moreover, shVEGF-C more significantly enhanced bladder cancer cell sensitivity to cisplatin compared to the VEGF-C control (Fig. 6A, 6B).

## Discussion

Emerging evidence shows that miR-101 expression is lost in bladder cancer tissues and bladder cancer cell lines [15,31]. The NDY1/KDM2B-miR-101-EZH2 axis has been identified as active in bladder cancer cells [32], wherein miR-101 suppresses the motility of T24 bladder cancer cells by targeting c-Met’s 3′-UTR [33]. However, the detailed molecular mechanism(s) of miR-101’s role in bladder cancer cells remains to be elucidated. Therefore, by utilizing a TargetScan-based bioinformatic approach to search for potential miR-101 targets, we identified VEGF-C as a potential miR-101 target. We then constructed a miR-101-overexpressing lentivirus for transfection into the T24 and 5637 bladder cancer cell lines and demonstrated that miR-101 overexpression inhibits bladder cancer cell migration and invasion. Moreover, we demonstrated that miR-101 negatively regulates VEGF-C protein expression, and VEGF-C overexpression rescues the effects of miR-101 overexpression, suggesting that miR-101 negatively regulates VEGF-C expression post-transcriptionally. Finally, we showed that both miR-101 and VEGF-C interference independently enhanced cisplatin cytotoxicity in bladder cancer cells, and VEGF-C also rescued miR-101’s effect on bladder cancer cell sensitivity to cisplatin.

The production and secretion of VEGFs—potent stimulators of angiogenesis that affect endothelial cell proliferation and motility as well as vascular permeability—has been commonly observed in several aggressive tumor types and significantly influences the prognosis of cancer patients [28], Although VEGF-C has been shown to have angiogenic effects on endothelial cells [34], VEGF-C primarily acts lymphatically through the Flt4/VEGFR-3 and KDR/VEGFR-2 receptor tyrosine kinases [35], which are exclusively expressed in the lymphatic endothelium and high venular endothelium of lymph nodes in normal adult tissues. For example, VEGF-C overexpression has been shown to produce lymphatic endothelial proliferation and selective hyperplasia of the lymphatic vasculature [36].

With respect to VEGF-C’s role in malignancy, VEGF-C mRNA expression was initially detected in a mixed set of solid human tumors with VEGF-C’s role in lymphangiogenesis providing a possible mechanism for metastasis via newly-formed lymphatic vessels [37]. Later studies discovered that VEGF-C’s primary receptor Flt4/VEGFR-3 is expressed in a variety of human malignancies, including lung, colorectal, prostate, and squamous cell carcinomas of the head and neck as well as Kaposi’s sarcoma [28]. Moreover, positive correlations have been found between VEGF-C expression and lymphatic spread in bladder TCC [38] as well as in prostate [39], colorectal [40], gastric [41], thyroid [42], and neuroblastoma cancers [43]. VEGF-C downregulation reduces murine lung and colon cancer metastases, and Flt4/VEGFR-3 inhibition has been associated with reduced angiogenesis/lymphangiogenic activities as well as reduced growth and metastasis in breast, pancreatic, and ovarian cancers, paving the way for the development and testing of VEGFR3 inhibitors as targeted chemotherapeutics [44]. Here, miR-101 overexpression repressed VEGF-C protein expression, impairing bladder cancer cell migration and invasion *in vitro* in the absence of lymphangiogenesis. These findings suggest that VEGF-C acts through non-lymphangiogenic mechanism(s) in promoting bladder tumor progression. Further research is required to determine the downstream affector(s) of the VEGF-C-Flt4/VEGFR-3 axis in bladder cancer cells that promote bladder cancer cell mobility.

Cisplatin chemotherapy remains the first-line treatment for locally advanced bladder cancer, so cisplatin resistance is a major clinical issue for bladder cancer patients [45]. Although we found no previous work investigating miR-101’s role on cisplatin resistance in bladder cancer cells, miR-101 expression has been shown to be downregulated in docetaxel-induced cisplatin cross-resistant head and neck squamous cell carcinoma cell lines [46], while miR-101 has been shown to synergize with cisplatin, doxorubicin, and fluorouracil in inducing apoptosis in hepatocellular carcinoma cells [47,48]. As miR-101’s antagonist, VEGF-C promotes cisplatin resistance in gastric cancer cells [29], and VEGF-C upregulation results in tumor resistance to anti-VEGF therapy in a murine lung cancer model [49]. In agreement with these previous findings, here both miR-101 and VEGF-C interference were shown to independently enhance cisplatin cytotoxicity in bladder cancer cells, and VEGF-C also rescued miR-101’s effect on bladder cancer cell sensitivity to cisplatin. However, the mechanism(s) underlying VEGF-C’s role in the cisplatin resistance of bladder cancer cells remains unknown and requires further study.

There are two limitations to this study. First, this study shows that VEGF-C plays a key role in bladder cancer cell migration, invasion, and cisplatin chemosensitivity and is a direct target of miR-101. However, this fact does not preclude the possibility that miR-101 has other targets that have similar cellular functions to VEGF-C. Using the TargetScan database, we found 803 transcripts that are predicted to be targets of hsa-miR-101, including EZH2, BTBD3, NLK, and MITF. Further study is needed to determine whether these 803 putative targets are actual targets of miR-101 and whether any such protein targets are associated with bladder cancer cell migration, invasion, and cisplatin chemosensitivity. Second, previous work has shown that miR-101 is downregulated in TCC and that miR-101 inhibits TCC cell proliferation and colony formation through directly repressing the histone methyltransferase EZH2 [15]. Therefore, in this study, we did not assess miR-101’s effects upon bladder cancer cell proliferation but rather specifically focused on miR-101’s influence on bladder cancer cell migration and invasion through VEGF-C. Thus, whether or not the miR-101-VEGF-C axis affects bladder cancer cell proliferation remains an open question. Third, the transfection efficiency of miR-101 lentivirus was 99%, but the transfection efficiency of the VEGF-C plasmid was only 40%; due to technical photographic issues, we assumed that all cells were miR-101+ (GFP+) and thereby selected cells by FACS solely on the basis of VEGF-C+ (RFP+). In short, VEGF-C+ (RFP+) cells were deemed double-positive cells. Due to this technical limitation, it was difficult to appropriately assess co-localization of miR-101 and VEGF-C mRNA.

In summary, we demonstrate miR-101 post-transcriptionally suppresses VEGF-C expression, inhibits bladder cancer cell migration and invasion, and increases cisplatin sensitivity. Although further research is needed to identify other relevant targets of miR-101, this study provides new insight into miR-101’s role in bladder cancer and shows miR-101’s promise as a potential molecular target for bladder cancer.

## Supporting Information

S1 Data The Construction of miR-101-overexpression Plasmid and VEGF-C-overexpression Plasmid (DOC)

S1 Fig Representative Image of Bladder Cancer T24 Cells Simultaneously Transfected with miR-101/green fluorescent Protein (miR-101/GFP) and VEGF-C/red fluorescent protein (VEGF-C/RFP) (TIF)

S1 Table Firely/Renilla Luciferase Analyses of the Transfection with wt and mut VEGF-C 3′UTR and Either miR-101-overexpressing Vector or a Scrambled-sequence Vector. (XLSX)
