# Supplementary material for: miR-101 Suppresses Vascular Endothelial Growth Factor C That Inhibits Migration and Invasion and Enhances Cisplatin Chemosensitivity of Bladder Cancer Cells
Source: PLoS One. 2015 Feb 6;10(2):e0117809. doi: 10.1371/journal.pone.0117809 (PMC4320037; doi:10.1371/journal.pone.0117809)
Supplement: S1 Data — (DOC) [file pone.0117809.s001.doc]

**1. The Construction of miR-101-overexpression Plasmid**

**
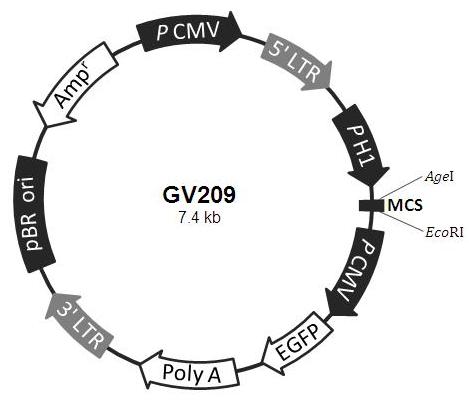
**

**Obtain the miR-101 targeted fragment**

**Primer:**

| hsa-mir-101-P1 | CGGGTACCGGTAGTCCTTCACTTCATGGGGAG |
| --- | --- |
| hsa-mir-101-P2 | CGGAATTCAAAAAACCCAGCCACCTGTTTCAC |

**Sequence and Blast (positive clone)**

GaATTACAAAAACAAATTACAAAAATTCAAAATTTTCGGGTTTATTACAGGGACAGCAGAGATCCAGTTTGGTTAGTACCGGGCCCGCTCTAGACTCGAGATATTTGCATGTCGCTATGTGTTCTGGGAAATCACCATAAACGTGAAATGTCTTTGGATTTGGGAATCTTATAAGTTCTGTATGAGACCACTCACCGGTAGTCCTTCACTTCATGGGGAGCCTTCAGAGAGAGTAATGCAGCCACCAGAAAGGATGCCGTTGACCGACACAGTGACTGACAGGCTGCCCTGGCTCAGTTATCACAGTGCTGATGCTGTCTATTCTAAAGGTACAGTACTGTGATAACTGAAGGATGGCAGCCATCTTACCTTCCATCAGAGGAGCCTCACCGTACCCAGGAAGAAAGAAGGTGAAAGAGGAATGTGAAACAGGTGGCTGGGTTTTTTGAATTCGGATCCATTAGGCGGCCGCGTGGATAACCGTATTACCGCCATGCATTAGTTATTAATAGTAATCAATTACGGGGTCATTAGTTCATAGCCCATATATGGAGTTCCGCGTTACATAACTTACGGTAAATGGCCCGCCTGGCTGACCGCCCAACGACCCCCGCCCATTGACGTCAATAATGACGTATGTTCCCATAGTAACGCCAATAGGGACTTTCCATTGACGTCAATGGGTGGAGTATTTACGGTAAACTGCCCACTTGGCAGTACATCAAGTGTATCATATGCCAAGTACGCCCCCTATTGACGTCAATGACGGTAAATGGCCCGCCTGGCATTATGCCCAGTACATGACCTTATGGGACTTTCCTACTTGGCAGTACATCTACGTATTAGTCATCGCTATTACCATGGTGATGCGGTTTTGGCAGTACATCAATGGGCGTGGATAGCGGTTTGACTCACGGGGATTTCCAAGTCTCCACCCCATTGACGTCAATGGAGTTTGTTTTGGCACCAAAATCAACGGACTTTCCAAAATGTCGTAACACTCCGCCCCATTGACGCAAATGGGCGGTAGGCGTGTACGGTGGGAGGTCTTATATAAGCAGGAGCGTGTTTGGTGACGTCAGAATCCGCTAGGCGCTACGGGACGCCACCATGGTGAGCCAGGGCGAGGAGCTGATCACTGGGTTGGTGCCCATCATGACTGACTGGAACGGCCTACGTAAACGGCACCAAGTTCTACGTGTGTCCGAGCGC

**2. The Construction of VEGF-C-overexpression Plasmid**

**
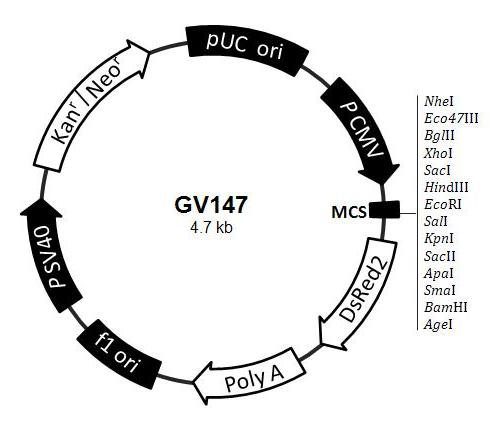
**

**Obtain the VEGF-C target fragment**

**Primer**

| VEGFC-P1 | TCCGCTCGAGATGCACTTGCTGGGCTTCTTC |
| --- | --- |
| VEGFC-P2 | ATGGGGTACCGTGCTCATTTGTGGTCTTTTCC |

**Sequence and blast:**

CTGGTTTAGTGACCGTCAGATCCGCTAGCGCTACCGGACTCAGATCTCGAGATGCACTTGCTGGGCTTCTTCTCTGTGGCGTGTTCTCTGCTCGCCGCTGCGCTGCTCCCGGGTCCTCGCGAGGCGCCCGCCGCCGCCGCCGCCTTCGAGTCCGGACTCGACCTCTCGGACGCGGAGCCCGACGCGGGCGAGGCCACGGCTTATGCAAGCAAAGATCTGGAGGAGCAGTTACGGTCTGTGTCCAGTGTAGATGAACTCATGACTGTACTCTACCCAGAATATTGGAAAATGTACAAGTGTCAGCTAAGGAAAGGAGGCTGGCAACATAACAGAGAACAGGCCAACCTCAACTCAAGGACAGAAGAGACTATAAAATTTGCTGCAGCACATTATAATACAGAGATCTTGAAAAGTATTGATAATGAGTGGAGAAAGACTCAATGCATGCCACGGGAGGTGTGTATAGATGTGGGGAAGGAGTTTGGAGTCGCGACAAACACCTTCTTTAAACCTCCATGTGTGTCCGTCTACAGATGTGGGGGTTGCTGCAATAGTGAGGGGCTGCAGTGCATGAACACCAGCACGAGCTACCTCAGCAAGACGTTATTTGAAATTACAGTGCCTCTCTCTCAAGGCCCCAAACCAGTAACAATCAGTTTTGCCAATCACACTTCCTGCCGATGCATGTCTAAACTGGATGTTTACAGACAAGTTCATTCCATTATTAGACGTTCCCTGCCAGCAACACTACCACAGTGTCAGGCAGCGAACAAGACCTGCCCCACCAATTACATGTGGAATAATCACATCTGCAGATGCCTGGCTCAGGAAGATTTTATGTTTTCCTCGGATGCTGGAGATGACTCAACAGATGGATTCCATGACATCTGTGGACCAAACAAGGAGCTGGATGAAGAGACCTGTCAGTGTGTCTGCAGAGCGGGGCTTCGGCCTGCCAGCTGTGGACCCCACAAAGAACTAGACAGAAACTCATGCCAGTGTGTCTGTAAAAACAAACTCTTCCCCAGCCAATGTGGGGCCAACCGAGAATTTGATGAAAACACATGCCAGTGTGTATGTAAAAGAACCTGCCCCAGAAATCAACCCCTAAATCCTGGAAAATGTGCCTGTGAATGTACAGAAAGTCCACAGAAATGCTTGTTAAAAGGAAAGAAGTTCCACCACCAAACATGCAGCTGTTACAGACGGCCATGTACGAACCGCCAGAAGGCTTGTGAGCCAGGATTTTCATATAGTGAAGAAGTGTGTCGTTGTGTCCCTTCATATTGGAAAAGACCACAAATGAGCACGGTACCGCGGGCCCGGGATCCACCGGTCGCCACCATGGTGCGCTCCTCCAAGAACGTCATCAAGGAGTTCATGCGCTTCAAGG
