# Supplementary figures and images for: miR-101 Suppresses Vascular Endothelial Growth Factor C That Inhibits Migration and Invasion and Enhances Cisplatin Chemosensitivity of Bladder Cancer Cells
Source: PLoS One. 2015 Feb 6;10(2):e0117809. doi: 10.1371/journal.pone.0117809 (PMC4320037; doi:10.1371/journal.pone.0117809)

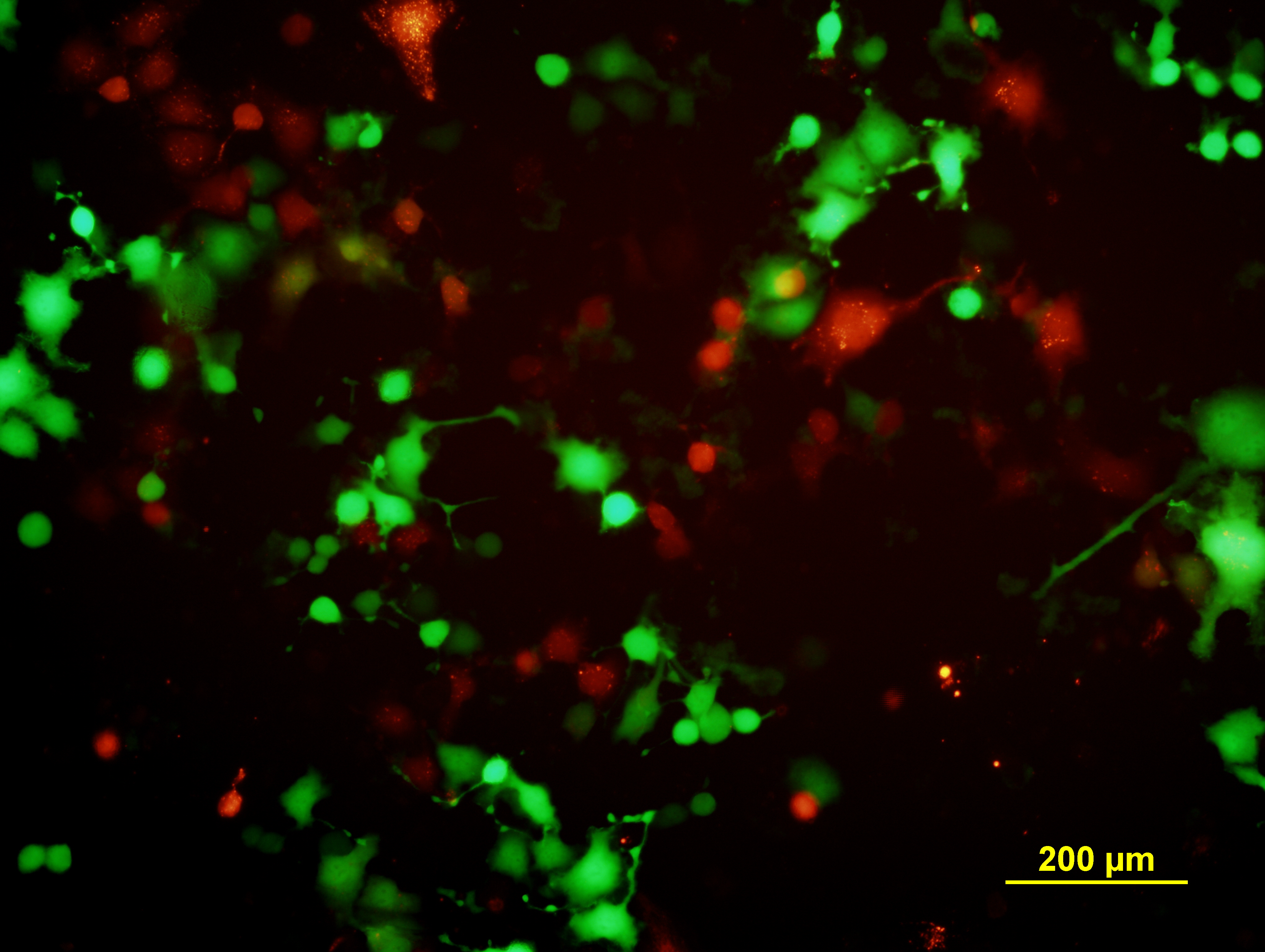

Supplement: S1 Fig — (TIF) [file pone.0117809.s002.tif]
